# Risk factors of vesicoureteral reflux and urinary tract infections in children with imperforate anus: A population-based case-control study in Taiwan

**DOI:** 10.1097/MD.0000000000027499

**Published:** 2021-11-05

**Authors:** Chung-Wei Wu, Chang-Ching Wei, Cheng-Li Lin, Hsiao-Huei Chao, Tse-Chun Wei, Tsung-Hsueh Hsieh, Ching-Yuang Lin

**Affiliations:** a Division of Pediatric Nephrology, Children's Hospital of China Medical University, Taichung, Taiwan; b School of Medicine, China Medical University, Taichung, Taiwan; c Management Office for Health Data, China Medical University Hospital, Taichung, Taiwan; d Institute of Biostatistics, China Medical University, Taichung, Taiwan; e Division of Pediatric Surgery, Children's Hospital of China Medical University, Taichung, Taiwan; f Department of Pediatrics, Chung Shan Medical University Hospital, Taichung, Taiwan.

**Keywords:** congenital anomaly of the kidney and urinary tract, imperforate anus, urinary tract infection, vesicoureteral reflux

## Abstract

Imperforate anus (IA) is associated with several urological anomalies, including vesicoureteral reflux (VUR), a major contributor to high morbidity in patients with anorectal malformations. This retrospective study was performed to elucidate the risk factors of vesicoureteral reflux (VUR) and UTI in children with IA.

We used the National Health Insurance Research Database (NHIRD) to estimate the frequency of congenital anomalies of the kidney and urinary tract (CAKUT) in children with IA. We also investigated the frequencies of VUR, UTI, and CAKUT in children with IA along with the risk factors of VUR.

We enrolled 613 children between 2000 and 2008 (367 males and 246 females; 489 low-position IA and 124 high-position IA). High-position IA was associated with a significantly increased risk of VUR compared with low-position IA (OR: 2.68, 95% CI: 1.61, 4.45). In addition, children with IA along with CAKUT, hydronephrosis, or UTI had a higher risk of VUR (OR: 8.57, 95% CI: 3.75, 19.6; OR: 7.65, 95% CI: 4.48, 13.1; and OR: 31.8, 95% CI: 11.5, 88.3, respectively). UTI, as well as chromosomal anomalies, were more frequent in children with high-position IA.

Patients with a high-position IA had a greater risk of VUR, particularly those with CAKUT, hydronephrosis, or UTI. Such patients must periodically undergo urinalysis to screen for UTI and early voiding cystourethrogram to rule out VUR and prevent consequent renal damage. Chromosomal analysis is suggested to rule out Down syndrome.

## 1 Introduction

Imperforate anus (IA), an anorectal malformation, is a rare congenital anomaly found in the lowest portion of the intestinal and urogenital tracts. The incidence of anorectal malformations varies from 2–2.5 per 10,000 live births, with considerable variations across regions worldwide.^[1,2]^ The concept of caudal repression may apply to anogenital anomalies of the anus, which might predict a higher incidence of anomalies of the lower spine and genitourinary tract in association with IA.^[3]^ Previously, IA was clinically classified into high and low subtypes, depending on whether the distal rectal pouch ended above or below the levator muscle level.^[3]^ High anomalies are characterized by anorectal agenesis or rectal atresia.^[4]^ In the high-position, the rectal pouch does not enter the puborectalis muscle. In the low-position, the rectum passes through a well-developed puborectalis muscle. Associated anomalies are twice more common in the high-position than those in the low-position variety.^[5]^

Congenital anomalies of the urinary tract are the significant anomalies associated with anorectal malformations.^[3]^ Anorectal malformations have a high incidence of associated genitourinary tract anomalies, with the risk of renal failure ranging from 25–50%.^[6–8]^ Vesicoureteral reflux (VUR) has been reported in 20–47% of children with anorectal malformations.^[9]^ The presence of VUR increases the risk of developing febrile urinary tract infections (UTI), leading to renal scarring and subsequent renal dysfunction.^[10,11]^ Most genital anomalies can be detected by clinical examination; however, certain urological anomalies, which are a major contributory factor to high morbidity in children with anorectal malformations, require further clinical investigation. Therefore, this study aimed to explore the frequency of congenital anomalies of the kidney and urinary tract (CAKUT) and to investigate the risk factors of vesicoureteral reflux (VUR) in children with IA (both low and high positions). Understanding the risk of VUR and its association with UTI may be beneficial for the early diagnosis of this condition, thereby preventing consequent renal damage.

## 2 Patients and methods

### 2.1 Data source

This was a retrospective, population-based study using claims records from the Taiwan National Health Insurance Research Database (NHIRD). The NHIRD has collected claims records covering all inpatient and outpatient medical benefit claims close to the entire population of Taiwan since the inception of its single-payer National Health Insurance (NHI) program in 1995. The NHI covers nearly 99% of Taiwan's population. In Taiwan, the NHIRD is a publicly available database through formal application and approval by the Health and Welfare Data Science Center of the Ministry of Health and Welfare (http://dep.mohw.gov.tw/DOS/np-2500-113.html).^[11]^

### 2.2 Ethics

In line with the Personal Information Protection Act, the information was deidentified before providing the dataset to the researchers; thus, informed consent was not required for this study. This study was approved by the Institutional Review Board of the China Medical University Hospital (CRREC-103-048). The International Classification of Disease, Ninth Revision, Clinical Modification (ICD-9-CM) was used for diagnosis.

### 2.3 Study participants

We selected all subjects <18 years of age who were diagnosed with IA with an ICD-9-CM code of 751.2 in any diagnosis field of inpatient claims or ambulatory claims between 2000 and 2012. High-position malformations were identified by emergent colostomy (ICD-9-CM code, 46.1), followed by a pull-through operation during infancy, while low-position malformations were identified by neonatal perineoplasty without colostomy. Hence, a high-position IA was defined according to the ICD-9-CM codes 751.2 and 46.1, while a low-position IA was defined according to the ICD-9-CM code 751.2 (without 46.1). From the selected subjects, we identified those with a diagnosis of CAKUT, including renal agenesis and dysgenesis (ICD-9-CM code, 753.0), renal dysplasia (ICD-9-CM code, 753.15), ureteral obstruction [ICD-9-CM code, 753.2 Obstructive defects of renal pelvis and ureter; including 753.20 (Unspecified obstructive defects of renal pelvis and ureter), 753.21 (Congenital obstruction of the ureteropelvic junction), 753.22 (Congenital obstruction of the ureterovesical junction), 753.23 (Congenital ureterocele), and 753.29 (Other).], VUR (ICD-9-CM code, 593.7), hydronephrosis (ICD-9-CM code, 591), and other ureter and bladder anomalies (ICD-9-CM code, 753.3–753.6). Primary VUR was identified as subjects with VUR but without neurogenic bladder (ICD-9-CM codes, 596.4, 596.5, and 344.61). We also identified the UTI diagnosis in all study subjects’ claim records using ICD-9-CM codes 590.1, 590.2, 590.8, 590.9, 595.9, 599.0, and 996.64. The chromosomal analysis was selected by ICD 9 code **758** (Chromosomal anomalies, including 758.0 Down's syndrome, 758.1 Patau's syndrome, 758.2 Edward's syndrome, 758.3 Autosomal deletion syndromes, 758.4 Balanced autosomal translocation in a normal individual, 758.5 Other conditions due to autosomal anomalies, and others).

### 2.4 Statistical analyses

The continuous data are expressed as the mean (standard deviation), and the categorical variables are represented by percentages. A chi-square test was used to analyze the categorical variables. The t-test was used to examine differences for continuous variables. The multivariable unconditional logistic regression analysis was used to calculate odds ratios (ORs) and 95% confidence intervals (95% CIs) for the association between risk factors and VUR. Risk factors such as sex, type of IA, CAKUT (except VUR), Hydronephrosis, ever UTI, chromosomal anomaly, high-position of IA and CAKUT (except VUR), high-position of IA and CAKUT (except VUR), and ever UTI were included in the multivariable unconditional logistic regression model and analyzed multiple times. All statistical analyses were performed using SAS software, version 9.1. A *P*-value of < .05 was considered statistically significant.^[12]^

## 3 Results

### 3.1 Clinical characteristics of the study subjects

A total of 613 subjects [367 (59.8%) males and 246 (40.1%) females] were diagnosed with IA. Among them, 489 (198 females and 291 males) patients had a low-position IA, and 124 (48 females and 76 males) had a high-position IA.

### 3.2 Incidence of CAKUT in patients with IA

To understand the possible association between CAKUT and IA, we evaluated the incidence of major urinary tract anomalies in patients with anorectal malformations between 2000 and 2012. Table 1 depicts the clinical characteristics of children with IA. The five most common urinary tract anomalies in patients with IA were VUR, hydronephrosis, renal agenesis, ureteral obstruction, and cystic dysplastic kidney. Children with a high-position IA had a higher incidence of VUR, hydronephrosis, UTI, and Down syndrome than those with a low-position IA.

**Table 1:** Clinical characteristics of children with imperforate anus.

	Imperforate anus		
	Low-position	High-position	
	N=489	N=124	
	n (%)	n (%)	*P*-value
Age (years), mean (SD)^∗^	2.45 (3.91)	2.28 (2.98)	.65
Sex			.72
Female	198 (40.5)	48 (38.7)	
Male	291 (59.5)	76 (61.3)	
CAKUT(except VUR)	71 (14.5)	30 (24.2)	.095
Renal agenesis and dysgenesis	10 (2.0)	3 (2.4)	.73
Renal dysplasia	1 (0.2)	0 (0.0)	–
Obstruction of ureter	6 (0.1.2)	0 (0.0)	–
Hydronephrosis	58 (11.9)	24 (19.4)	**.03**
Others	3 (0.6)	1 (0.8)	.41
Vesicoureteral reflux	50 (10.2)	29 (23.4)	**< .001**
Primary	15 (3.1)	9 (7.3)	**.03**
Secondary	35 (7.2)	20 (16.1)	**.002**
Urinary tract infection	238 (48.7)	91 (73.4)	**< .001**
Neurogenic bladder	43 ( 8.79)	14 ( 11.29)	.39
Atonic neurogenic bladder	1 (0.2)	0 (0.0)	–
CES with neurogenic bladder	16 (3.3)	5 (4.0)	.68
Others	26 (5.3)	9 (7.3)	.41
Chromosomal anomaly	13 (2.7)	9 (7.3)	**.01**
Down syndrome	3 (0.6)	6 (4.8)	**.003**

Chi-square test was performed.

∗ *t*-test was performed. CAKUT(except VUR) = congenital anomalies of the kidney and urinary tract excepted those with vesicourethral reflux, CES = cauda equina syndrome.

### 3.3 Risk of VUR in children with IA

Table 2 demonstrates the risk of VUR stratified by the clinical characteristics of the patients. High-position IA was associated with a significantly increased risk of VUR compared with low-position IA (OR: 2.68, 95% CI: 1.61, 4.45). In addition, children with IA along with CAKUT, hydronephrosis, or UTI had a higher risk of VUR (OR: 8.57, 95% CI: 3.75, 19.6; OR: 7.65, 95% CI: 4.48, 13.1; and OR: 31.8, 95% CI: 11.5, 88.3, respectively). When we differentiated the high- and low-position varieties of IA in the analysis, children with IA and UTI had the highest risk of VUR (high-position IA: OR: 29.8, 95% CI: 39.0, 228.2 (Table 3); low-position IA: OR: 29.6, 95% CI: 9.06, 96.6 (Table 4)). In patients with high-position IA, UTI had the highest risk of VUR, followed by hydronephrosis and CAKUT. In patients with low-position IA, UTI had the highest risk of VUR, followed by CAKUT and hydronephrosis.

**Table 2:** Factors associated with vesicoureteral reflux in children with imperforate anus.

Variable	OR (95% CI)^∗^	*P*-value
Gender		
Female	1 (Reference)	
Male	1.34 (0.82, 2.20)	.42
Type of imperforate anus		
Low position	1 (Reference)	
High position	2.68 (1.61, 4.45)	< .001
CAKUT(except VUR)		
No	1 (Reference)	
Yes	8.57 (3.75, 19.6)	< .001
Hydronephrosis		
No	1 (Reference)	
Yes	7.65 (4.48, 13.1)	< .001
UTI		
No	1 (Reference)	
Yes	31.8 (11.5, 88.3)	< .001
Chromosomal anomaly		
No	1 (Reference)	
Yes	1.07 (0.31, 3.70)	.73
High position of IA and CAKUT(except VUR)		
No	1 (Reference)	
Yes	14.2 (2.55, 78.8)	< .001
High position of IA and CAKUT(except VUR) and UTI		
No	1 (Reference)	
Yes	28.4 (3.14, 257.7)	< .01

∗ Adjusted for sex and age. 95% CI = 95% confidence intervals, CAKUT(except VUR) = congenital anomalies of the kidney and urinary tract excepted those with vesicourethral reflux, IA = imperforate anus, OR = odds ratios, UTI = urinary tract infection.

**Table 3:** Factors associated with vesicoureteral reflux in children with imperforate anus of high- position varieties.

	Imperforate anus			
	High-position			
Variable	OR^∗^	(95% CI)		
Gender (male vs female)	1.55	(0.64, 3.76)		
CAKUT(except VUR) (present vs absent)	7.44	(1.29, 43.0)^†^		
Hydronephrosis (present vs absent)	13.4	(4.78, 37.5)^§^		
UTI (present vs absent)	29.8	(3.90, 228.2)^‡^		
Infection of kidney (present vs absent)	31.1	(4.07, 238.0)^‡^		
Chromosomal anomaly (present vs absent)	0.93	(0.18, 4.75)		
CAKUT(except VUR) and UTI (present vs absent)	15.0	(1.61, 140.6)^§^	–	–

∗ Adjusted for sex and age.

† *P* < .05.

‡ *P* < .01.

§ *P* < .001. 95% CI = 95% confidence intervals, CAKUT (except VUR) = congenital anomalies of the kidney and urinary tract excepted those with vesicourethral reflux, IA = imperforate anus, OR = odds ratios, UTI = urinary tract infection.

**Table 4:** Factors associated with vesicoureteral reflux in children with imperforate anus of low-position varieties.

	Imperforate anus	
	Low-position	
Variable	OR^†^	(95% CI)
Gender (male vs female)	1.55	(0.64, 3.76)
CAKUT(except VUR) (present vs absent)	9.42	(3.62, 24.5)^∗∗∗^
Hydronephrosis (present vs absent)	5.61	(2.89, 10.9)^∗∗∗^
UTI (present vs absent)	29.6	(9.06, 96.6)^∗∗∗^
Infection of kidney (present vs absent)	22.6	(7.99, 64.0)^∗∗∗^
Chromosomal anomaly (present vs absent)	0.73	(0.09, 5.71)

† Adjusted for sex and age. ^∗^*P* < .05. ^∗∗^*P* < .01.

∗∗∗ *P* < .001. 95% CI = 95% confidence intervals, CAKUT (except VUR) = congenital anomalies of the kidney and urinary tract excepted those with vesicourethral reflux, IA = imperforate anus, OR = odds ratios, UTI = urinary tract infection.

### 3.4 Frequency of chromosomal abnormalities

There is a genetic component of IA. We found that patients with a high-position IA had a significantly (*P* = .01) higher incidence of chromosomal anomalies than those with a low-position IA (Table 1), which was more predominant in patients with Down syndrome. We found that high type IA showed a higher frequency of chromosomal anomaly than low type (7.3% vs 2.7%), particularly of Down syndrome, which suggested a focus on Down's syndrome (4.8% vs 0.6%). However, in our study, we did not find an association between chromosomal anomaly and increased risks of VUR. Further, there was no difference in the incidence of VUR and UTI between patients with high- or low-position IA (Tables 2 and 3).

### 3.5 End-stage renal disease

There was no progression to ESRD during our data collection periods.

## 4 Discussion

This population-based study was performed to investigate the frequency of CAKUT in children with IA and investigate the risk factors of VUR. The results consistently indicate that patients with a high-position IA had a greater risk of VUR, particularly those with CAKUT, hydronephrosis, or UTI. The four most common urinary tract abnormalities associated with high-position IA are unilateral agenesis, renal hypoplasia or dysplasia, VUR, and hydronephrosis.^[10,11,12]^ In contrast, the four most common urinary tract abnormalities associated with low-position IA are VUR, lower urinary tract abnormalities, duplication, and malrotation.^[10,13,14]^ In our study, the four most common urinary tract abnormalities of the high-position IA were hydronephrosis, renal agenesis, and VUR. The four most common urinary tract abnormalities associated with low-position IA were hydronephrosis, VUR, renal agenesis, and ureteral obstruction. Such patients must periodically undergo urinalysis to screen for UTI and early voiding cystourethrogram study to rule out VUR. The precautions can prevent consequent renal damage.

VUR is one of the most common urinary tract anomalies in children with IA,^[15]^ with an incidence rate of 20–47%.^[9]^ Children with a high-position IA have a higher prevalence of VUR (33–39%), while those with a low-position IA have a lower prevalence of VUR (20–37%).^[14,9]^ A retrospective study reported that >30% of children with IA had VUR, and 39% of these children developed febrile UTI.^[13]^ To the best of our knowledge, the current study is the largest study evaluating the association between VUR and IA thus far. We found that a high-position IA was associated with a greater risk of VUR, particularly in patients with hydronephrosis or UTI.

The development of UTI in the setting of VUR has been shown to significantly increase the risk of developing renal scarring and subsequent renal dysfunction.^[10,11]^ Many of these infections go undiagnosed in neonates and infants.^[8]^ Several studies have mentioned that patients with febrile UTI did not have VUR. According to the American Academy of Pediatrics guideline on the management of febrile UTI, VCUG should not be performed routinely after the first episode of febrile UTI, and VCUG is indicated if renal ultrasonography reveals abnormal findings, such as hydronephrosis, renal scarring, or other findings suggestive of high-grade VUR or obstructive uropathy.^[16]^ However, it remains unclear whether the scope of this guideline can be expanded to include children with IA. Our study provides data on VUR in children with IA. We found that the high-position IA was significantly associated with UTI. However, another review has mentioned that VUR is the most common abnormality in patients with a low-position IA.^[14]^ Further, the symptoms in most patients with mild VUR resolved spontaneously with no apparent renal impairment.^[14]^ Several urological disorders are manifested and are noted only with the appearance of symptoms (e.g., neurogenic bladder) or deterioration of the clinical condition (e.g., renal failure secondary to VUR).

IA represents complex cloacal malformation. Mutations in a variety of different genes are likely to result in IA. An increased incidence of IA has been found in patients with Down syndrome.^[17]^ Similarly, in this study, we found an increased frequency of chromosomal anomalies in patients with IA. However, the correlation between chromosomal anomalies and IA requires further evaluation. The actual incidence of end-stage renal disease (ESRD) in patients with IA remains unknown. One small series analyzing 122 patients revealed a low incidence of only 0.8%.^[19]^ Aplastic or hypoplastic kidneys and VUR were the most common anomalies attributed to ESRD in the North American Pediatric Renal Trials and Collaborative Studies Database.^[19]^ In our study, there was no progression to ESRD in 12 years, perhaps due to the limited study period. Our results could not help determine the incidence of ESRD in patients with IA.

The present study was based on records generated by a highly accessible and well-used NHI system. The NHIRD is a nationwide, population-based database that includes all medical claims of >99% of the national population. Thus, the results were representative of the general population. Second, the database contains the original claims records of the studied population, and it was not designed for academic study; this attenuates bias in patient selection. There are several limitations to this study. First, it was a retrospective study with small sample size. Second, the NHIRD lacks important clinical data, such as grading of VUR, manifestations, laboratory data, and clinical outcomes. Third, the study did not discuss the clinical outcomes and treatment (ex: surgical rate and different surgical methods).

In conclusion, all patients with IA should be classified into high- or low-position IA. They should be evaluated for urogenital, spinal, and chromosomal anomalies to initiate early treatment. VCUG has been suggested in patients with IA and CAKUT or those with UTI for the first time. Concerning the radiation exposure of VCUG, contrast enhanced-VUS (ce-VUS) was discussed more recently due to radiation safety. Ce-VUS could be another method to consider.^[20]^ In patients with high-position IA, chromosomal analysis (e.g., fluorescence in situ hybridization) is required to rule out Down syndrome. These steps could prevent morbidity associated with a deteriorating renal function warranting salvage therapy.

## Acknowledgments

This study was based on data from the Center for Health and Welfare Data Science Center of the Ministry of Health and Welfare.

## Author contributions

**Conceptualization:** Chang-Ching Wei, Ching-Yuang Lin.

**Data curation:** Chang-Ching Wei, Cheng-Li Lin, Tse-Chun Wei, Ching-Yuang Lin.

**Formal analysis:** Chang-Ching Wei, Tse-Chun Wei.

**Methodology:** Chang-Ching Wei.

**Resources:** Chang-Ching Wei, Hsiao-Huei Chao.

**Writing – original draft:** Chung-Wei Wu, Tsung-Hsueh Hsieh, Ching-Yuang Lin.

**Writing – review & editing:** Chang-Ching Wei, Ching-Yuang Lin.
